# Optimization of High-Efficiency Tissue Culture Regeneration Systems in Gray Poplar

**DOI:** 10.3390/life13091896

**Published:** 2023-09-11

**Authors:** Huanhuan Li, Hang Wang, Lianke Guan, Zihui Li, Hua Wang, Jie Luo

**Affiliations:** College of Horticulture and Forestry Science, Hubei Engineering Technology Research Center for Forestry Information, Huazhong Agricultural University, Wuhan 430070, China; huanhuanli2022@163.com (H.L.); wangghangg@163.com (H.W.); lk_guan@163.com (L.G.); lizihui0428@163.com (Z.L.); near1981@mail.hzau.edu.cn (H.W.)

**Keywords:** poplar, regeneration in vitro, tissue culture, phytohormone, proliferation

## Abstract

A series of tissue culture regeneration protocols were conducted on gray poplar (*P. tremula × P. alba*) to select the most efficient callus induction medium, adventitious shoot differentiation medium, shoot elongation medium and rooting medium, which laid the foundation for the optimization of genetic transformation technology for gray poplar. The results showed that the Woody Plant Medium (WPM) supplemented with 0.10 mg L^−1^ kinetin (KT) and 1.00 mg L^−1^ 2,4-dichlorophenoxyacetic acid (2,4-D) was the most suitable medium for callus induction. The callus induction rates of different tissues were greater than 85.7%. The optimal adventitious shoot differentiation medium was the WPM supplemented with 0.02 mg L^−1^ thidiazuron (TDZ), and the adventitious shoot differentiation rates of young tissues were 22.2–41.9%. The optimal direct differentiation medium was the Murashige and Skoog (MS) medium supplemented with 0.20 mg L^−1^ 6-benzylaminopurine (6-BA), 0.10 mg L^−1^ indole butyric acid (IBA) and 0.001 mg L^−1^ TDZ, and the differentiation rate of adventitious shoots was greater than 94%. The best shoot elongation medium for adventitious shoots was the MS medium with 0.10 mg L^−1^ naphthylacetic acid (NAA). After 45 days of cultivation in the MS medium with 0.10 mg L^−1^ NAA, the average plant height was 1.8 cm, and the average number of elongated adventitious shoots was 11 per explant. The 1/2 MS medium with 0.10 mg L^−1^ NAA showed the best performance for rooting, and later, shoot growth. The direct shoot induction pathway can induce adventitious shoots much faster than the indirect adventitious shoot induction pathway can, and the time cost via the direct adventitious shoot induction pathway can be shortened by 2–6 weeks compared to that of the indirect shoot induction pathway.

## 1. Introduction

*Populus* is one of the most important afforestation tree species in China and an important woody plant for phytoremediation [1,2,3]. *Populus* is one of the fastest growing tree species, with high nutrient use efficiency and high wood production [4,5]. The wood from poplars is widely used for chopsticks and fiberboard, as well as energy materials [6,7,8]. With the completion of the whole genome sequencing of *Populus trichocarpa* and other poplar genotypes, poplar has become a model woody plant for studying the molecular biology of woody plants [9,10]. The establishment of a mature regeneration system is the basis for plant transformation and gene function study [11,12,13,14,15]. At present, plant tissue culture technology is becoming more and more mature, and many poplar genotypes have established mature regeneration systems [16,17,18]. The regeneration of many woody plants (including poplar and conifer species) can be carried out in various ways, such as those involving organs, somatic embryos and protoplasts, which lay the foundation for the genetic study of woody plants [6,7,19,20,21,22,23].

The main regeneration pathways include the direct regeneration of plant organs and the indirect regeneration of callus formation. Interestingly, there are many studies on the regeneration of poplar callus, and the regeneration efficiencies of different poplar genotypes are quite different [19,24]. For example, the callus differentiation rates of clones 064 (*P. trichocarpa × P. deltoides*), *P. tomentosa*, *P. davidiana × P. bolleana* and other genotypes obtained via callus regeneration are between 10% and 67% [24,25]. Although a callus is easy to produce, the induction cycle is long, and the process is complicated [26]. Compared with the indirect regeneration of a callus, the time cost for obtaining regenerated plants via direct organ regeneration is greatly lessened. Therefore, the direct organ regeneration pathway is also widely used in poplar [6,27,28,29,30].

Plant hormones play an important regulatory role in the processes of plant bud induction, proliferation and rooting [30,31]. There are obvious differences in the growth effects of different genotypes of poplar using different hormone types and ratios [32]. Plant hormones, such as 2,4-dichlorophenoxyacetic acid (2,4-D), indole butyric acid (IBA) and naphthylacetic acid (NAA), are commonly used as auxins in poplar tissue culture, which can promote cell division and elongation [26,30,33]. The ratio of auxin to cytokinin can be used to induce the formation, proliferation and rooting of adventitious shoots [25]. Cytokinins such as thidiazuron (TDZ), 6-benzyl aminopurine (6-BA), kinetin (KT) and other cytokinins are mostly used to promote cell division and differentiation, thereby promoting shoot regeneration [6,26,34]. The types and concentrations of these hormones have significant effects on the morphology and regeneration rate of explants [35]. Therefore, it is necessary to configure the plant growth regulators according to different culture requirements. At the same time, the tissue culture regeneration in poplar is also affected by many other factors, such as the genotype, growth status, explant type, growth environment and so on [36,37,38].

Poplar species are easier to transform and regenerate in vitro than other tree species are. Although many protocols have been developed for specific genotypes, the tissue culture regeneration schemes of poplar are not universal for most genotypes [19,27,33]. Gray poplar (*P. tremula × P. alba*) is sensitive to *Agrobacterium* and is a suitable poplar genotype for genetic transformation [39], with recently released complete genome information in the Phytozome database (*P. tremula × P. alba* v5.1, accessed on 1 August 2023, https://phytozome-next.jgi.doe.gov/). Recent studies also have demonstrated that gray poplar is an ideal poplar genotype for studying abiotic stresses in woody plants [40,41,42]. To obtain an efficient suitable regeneration protocol for gray poplar, a series of tissue culture regeneration protocols were conducted on gray poplar to select the most efficient callus induction medium, adventitious shoot differentiation medium, shoot elongation medium and rooting medium. The results obtained from this study will shorten the time cycle for the genetic transformation of gray poplar and can serve as a reference for other poplar genotypes.

## 2. Materials and Methods

### 2.1. Plant Culture

Gray poplar plants were in vitro maintained in Woody Plant Medium (WPM) with long-day conditions (16 h d^−1^) at 25 °C and sub-cultured every 2 months. The first six leaves (leaf interval index of 1–6) of the in vitro cultured plants were defined as young leaves, the corresponding stems were young stems, and the remaining parts were defined as old leaves and old stems. Each treatment was repeated three times.

### 2.2. Indirect Adventitious Shoot Induction

The main veins of the leaves from the culture plants tissues were cut with a scalpel, and the leaf edge and petiole were removed. The stem segments only retained the internodes, and the bud points were cut off. The wounded leaves and stems were then placed in two kinds of callus induction medium (CIM) to produce a callus: CIM1 (WPM + 0.50 mg L^−1^ KT + 1.00 mg L^−1^ 2,4-D) or CIM2 (WPM + 0.10 mg L^−1^ KT + 1.00 mg L^−1^ 2,4-D) [26]. The media were changed every 14 days until the callus was produced. The callus induction rates of different media were counted. After callus formation, they were transferred into the shoot induction medium (SIM), SIM1 (WPM + 0.02 mg L^−1^ TDZ) or SIM2 (WPM + 0.50 mg L^−1^ 6-BA + 0.05 mg L^−1^ NAA) [26], and the adventitious shoot differentiation rate of the callus was counted after 2–3 months.

### 2.3. Direct Induction of Adventitious Shoots

The treatment of explants was consistent with callus induction of adventitious shoots. The leaves and stem segments were placed in two different shoot induction media, SIM3 (Murashige and Skoog Medium (MS) + 0.20 mg L^−1^ 6-BA + 0.10 mg L^−1^ IBA + 0.001 mg L^−1^ TDZ) [6] or SIM4 (MS + 0.20 mg L^−1^ 6-BA +0.10 mg L^−1^ NAA + 0.01 mg L^−1^ TDZ) [8], to induce adventitious shoots. The media were changed every 14 days until adventitious shoots were differentiated. The differentiation rate of adventitious shoots in different media was counted after 1–2 months.

### 2.4. Shoot Elongation Medium

Adventitious shoots induced from stem segments in SIM3 were used as materials. Adventitious shoots were cultured in six different hormone combinations of shoot elongation media (SEM): SEM1 (MS + 0.10 mg L^−1^ IBA), SEM2 (MS + 0.10 mg L^−1^ IBA + 0.05 mg L^−1^ 6-BA), SEM3 (MS + 0.05 mg L^−1^ 6-BA), SEM4 (1/2 MS), SEM5 (WPM) or SEM6 (MS + 0.10 mg L^−1^ NAA) [43]. The number and height of adventitious shoots after 45 days were recorded.

### 2.5. Rooting Medium

**Figure 1:**
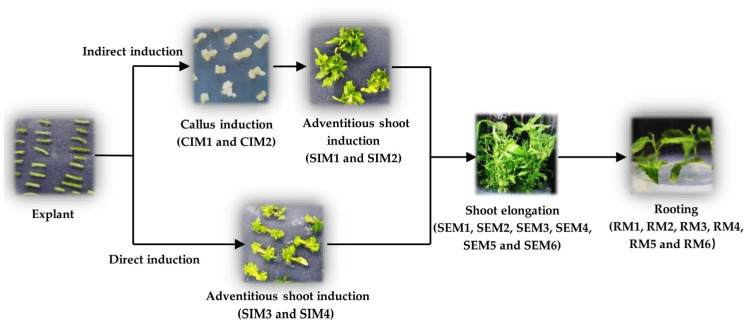
Flow chart of tissue culture regeneration processes for gray poplar. Callus induction medium (CIM): CIM1 (WPM + 0.50 mg L^−1^ KT + 1.00 mg L^−1^ 2,4-D) and CIM2 (WPM + 0.10 mg L^−1^ KT + 1.00 mg L^−1^ 2,4-D); shoot induction medium (SIM): SIM1 (WPM + 0.02 mg L^−1^ TDZ), SIM2 (WPM + 0.50 mg L^−1^ 6-BA + 0.05 mg L^−1^ NAA), SIM3 (MS + 0.20 mg L^−1^ 6-BA + 0.10 mg L^−1^ IBA + 0.001 mg L^−1^ TDZ) and SIM4 (MS + 0.20 mg L^−1^ 6-BA + 0.10 mg L^−1^ NAA + 0.01 mg L^−1^ TDZ); shoot elongation medium (SEM): SEM1 (MS + 0.10 mg L^−1^ IBA), SEM2 (MS + 0.10 mg L^−1^ IBA + 0.05 mg L^−1^ 6-BA), SEM3 (MS + 0.05 mg L^−1^ 6-BA), SEM4 (1/2 MS), SEM5 (WPM), and SEM6 (MS + 0.10 mg L^−1^ NAA); rooting medium (RM): RM1 (1/2 MS), RM2 (1/2 MS + 0.25 mg L^−1^ IBA), RM3 (1/2 MS + 0.10 mg L^−1^ NAA), RM4 (WPM), RM5 (WPM + 0.25 mg L^−1^ IBA) and RM6 (WPM + 0.10 mg L^−1^ NAA).

The adventitious shoots of tissue culture plantlets with two leaves and one bud with a length of about 1.5 cm were taken as experimental objects. Adventitious shoots were inserted into one of the following six rooting media (RM) with different hormone combinations: RM1 (1/2 MS), RM2 (1/2 MS + 0.25 mg L^−1^ IBA), RM3 (1/2 MS + 0.10 mg L^−1^ NAA), RM4 (WPM), RM5 (WPM + 0.25 mg L^−1^ IBA) or RM6 (WPM + 0.10 mg L^−1^ NAA). The plant height, number of roots, primary root length, and total fresh weight were recorded after 45 days. Detailed experimental information of the whole poplar tissue culture regeneration processes is shown in Figure 1. 

### 2.6. Data Statistics and Analysis

Analysis of variance (ANOVA) was performed, and the differences among treatments were considered statistically significant at *p*-value < 0.05 using Duncan’s multiple range tests. Statistical analyses were performed using SPSS statistical software (version 26.0, IBM Corporation, New York, NY, USA). Figures were drawn with Origin 2022 software (OriginLab Corporation, Northampton, MA, USA).

## 3. Results

### 3.1. Indirect Induction of Adventitious Shoots

**Figure 2:**
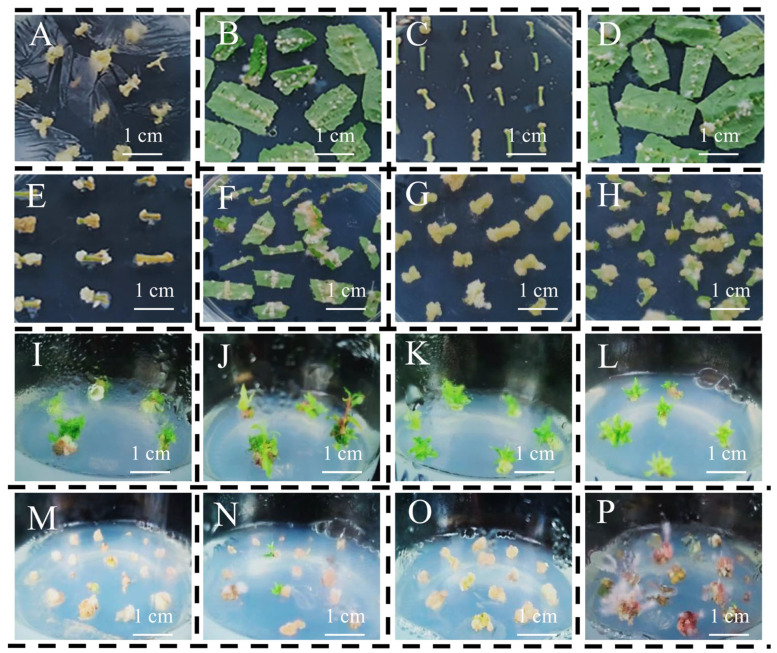
Comparison of the indirect regeneration pathways for the different tissues in gray poplar cultured on different media. (**A**): old stems after 29 days in CIM1; (**B**): old leaves after 29 days in CIM1; (**C**): young stems after 29 days in CIM1; (**D**): young leaves after 29 days in CIM1; (**E**): old stems after 29 days in CIM2; (**F**): old leaves after 29 days in CIM2; (**G**): young stems after 29 days in CIM2; (**H**): young leaves after 29 days in CIM2; (**I**): old stems after 69 days in SIM1; (**J**): old leaves after 69 days in SIM1; (**K**): young stems after 69 days in SIM1; (**L**): young leaves after 69 days in SIM1; (**M**): old stems after 69 days in SIM2; (**N**): old leaves after 69 days in SIM2; (**O**): young stems after 69 days in SIM2; (**P**): young leaves after 69 days in SIM2.

The four types of explants (i.e., old leaves, old stems, young leaves and young stems) were induced in CIM1 (Figure 2). After 29 days of cultivation in CIM1 and CIM2, the callus induction rates of the leaves reached 100% in both CIMs (Table 1 and Figure 2). The induction rates of the stem segments were slightly lower than those of the leaves, which were reduced by 7–20% in CIM1 compared to those of the leaves (Figure 2 and Table 1). The callus of the stem segments was mostly light yellow with a loose structure (Figure 2). The number of calluses induced from old stems and old leaves was higher than those from young tissues in CIM1 (Table 1 and Figure 2A–D).

After the callus induction in CIM1, the formed callus was transferred to SIM1 for further adventitious shoot induction. The callus began to form adventitious shoots on the 60th day, and the callus formed from a young stem showed a higher adventitious shoot induction ratio than those formed from other tissues (Table 1 and Figure 2I–L). The induction rates of adventitious shoots formed from the callus of a young stem and a young leaf were 41.9% and 22.2%, respectively (Table 1). The differentiation rates of adventitious shoots from the callus in the young parts were ca. 2–3 times higher than those in the old parts, and the differentiation rates of the callus from the stem segments were about 2 times larger than those from the leaves (Table 1). 

In CIM2, all types of explants had higher callus induction rates (Figure 2 and Table 1). Except for the callus induction rate of the old stem at 85.7%, the induction rates of the other three explants were all 100% (Table 1). Among the four tissues, young stems and young leaves more easily formed large and numerous calluses compared to those of the old tissues (Table 1 and Figure 2E–H). After being transferred to SIM2, the leaf callus changed from light yellow to light pink after 69 days of cultivation, and the individual calluses in the old leaves began to induce adventitious shoots (Figure 2M–P). About 3 months later, the callus from the leaves in SIM2 began to induce large numbers of adventitious shoots. The highest differentiation rate of the adventitious shoots reached 42.7% in the young leaves, while the differentiation rate of the old leaves was 20.8% (Table 1). However, the calluses induced from the stem segments did not differentiate into adventitious shoots (Table 1). These data suggest that SIM2 is not suitable for adventitious shoot induction using the calluses induced by the stem segments. 

### 3.2. Direct Induction of Adventitious Shoots

**Figure 3:**
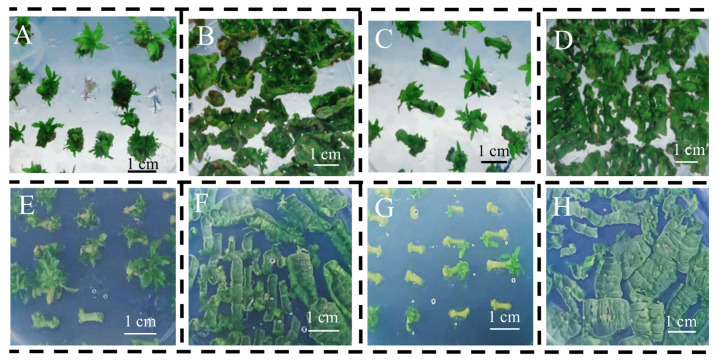
Comparison of the effects of direct regeneration pathways for different explants of gray poplar culturing 45 days in different media. (**A**): old stems in SIM3; (**B**): old leaves in SIM3; (**C**): young stems in SIM3; (**D**): young leaves in SIM3; (**E**): old stems in SIM4; (**F**): old leaves in SIM4; (**G**): young stems in SIM4; (**H**): young leaves in SIM4.

The explants in the two direct shoot induction media (SIM3 and SIM4) induced adventitious shoots after 45 days of culture (Table 2 and Figure 3). The leaves became crisp and curled, adventitious shoots formed from an incision in the veins, and adventitious shoots were induced at both ends of the stem segments in SIM3 and SIM4 (Figure 3). The leaves of the induced adventitious shoots in SIM3 had mostly expanded (Figure 3A–D), while some stem segments in SIM4 had not yet begun to differentiate adventitious shoots (Figure 3E,F). The number of adventitious shoots induced from stem segments was much greater than that induced from the leaves; only 1–3 adventitious shoots were formed from each bud point in the leaves (Figure 3). 

The differentiation rate of adventitious shoots in the young stems was about 95% in SIM3 and SIM4, and the differentiation rates of adventitious shoots in the other tissues reached 100% (Table 2). Compared to SIM4, the culture time of SIM3 was 7 days shorter than that of SIM4 (Table 2). 

### 3.3. Shoot Elongation Medium

**Figure 4:**
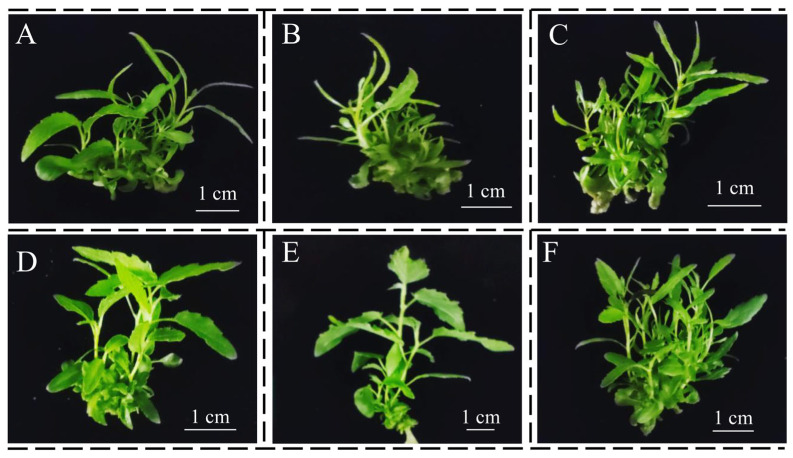
Comparison of adventitious shoot elongation in different shoot elongation media for 45 days. (**A**): SEM1 (MS + 0.10 mg L^−1^ IBA); (**B**): SEM2 (MS + 0.10 mg L^−1^ IBA + 0.05 mg L^−1^ 6-BA); (**C**): SEM3 (MS + 0.05 mg L^−1^ 6-BA); (**D**): SEM4 (1/2 MS); (**E**): SEM5 (WPM); (**F**): SEM6 (MS + 0.10 mg L^−1^ NAA).

**Figure 5:**
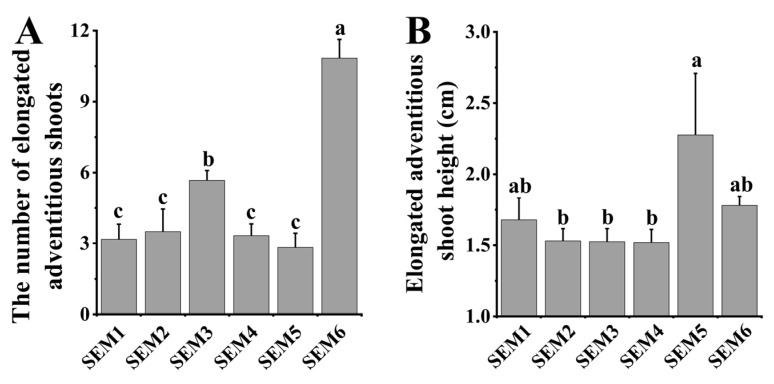
Effects of different shoot elongation media on the number (**A**) and height (**B**) of elongated adventitious shoots. Note: Different letters on the bars indicate significant differences. SEM1 (MS + 0.10 mg L^−1^ IBA), SEM2 (MS + 0.10 mg L^−1^ IBA + 0.05 mg L^−1^ 6-BA), SEM3 (MS + 0.05 mg L^−1^ 6-BA), SEM4 (1/2 MS), SEM5 (WPM) and SEM6 (MS + 0.10 mg L^−1^ NAA).

After cultivation in the different SEMs for 45 days, the number and height of elongated adventitious shoots (above 1 cm in height) were analyzed (Figure 4 and Figure 5). All the SEMs efficiently promoted the elongation of adventitious shoots, but the number and height of elongated adventitious shoots were significantly different among the six SEMs (Figure 4 and Figure 5).

In terms of the number of elongated adventitious shoots, SEM6 had the highest number of elongated adventitious shoots than the other SEMs did (Figure 4A). The average number of elongated adventitious shoots per stem segment was 11, which was significantly higher than those of the other media by 91.2–282.4% (Figure 5A). The number of elongated adventitious shoots in SEM3 was ca. 61.9% higher than those in SEM1, SEM2, SEM4 and SEM5 (Figure 4 and Figure 5A). There were no significant differences in the number of elongated adventitious shoots in SEM1, SEM2, SEM4 and SEM5, with an average number of about three elongated adventitious shoots (Figure 4 and Figure 5A).

In terms of plant height, only SEM5 was significantly different from the other five media, with an average plant height of 2.3 cm, which was ca. 27.7–49.7% higher than the other five media (Figure 4 and Figure 5B). This is mainly because SEM5 induced the roots to accelerate the absorption of nutrients and further increased the plant height (Figure 4E). SEM1 and SEM6 were grew slightly taller plants than SEMs 2–4 did by 9.8–17.2% (Figure 5B). Generally, SEM6 (MS with 0.10 mg L^−1^ NAA) had the highest number of elongated adventitious shoots with a desirable shoot height after 45 days of cultivation, which means it could be the best shoot elongation medium among the tested media for adventitious shoot elongation.

### 3.4. Rooting Medium

**Figure 6:**
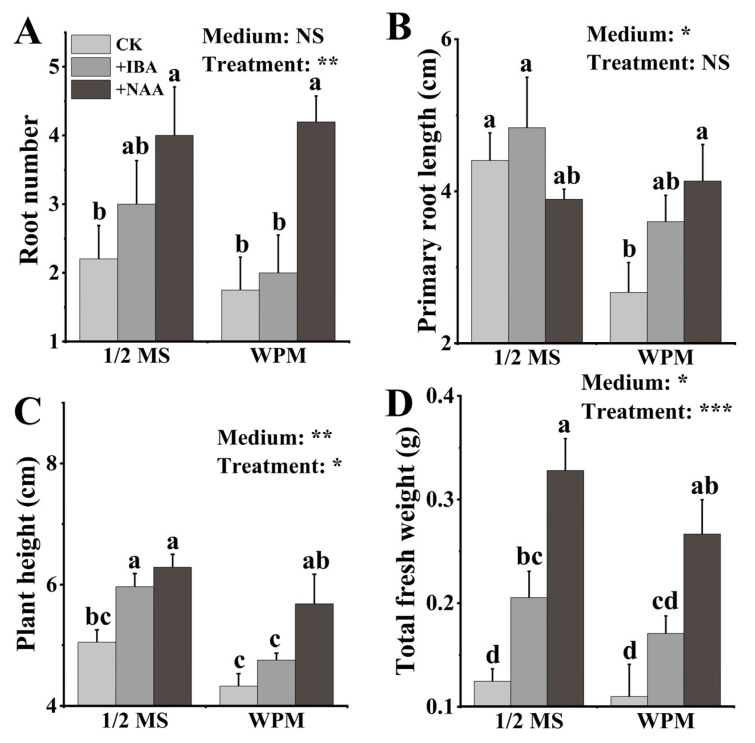
Growth of gray poplar in different rooting media (45 days). (**A**): the number of roots in different RMs; (**B**): the primary root length in different RMs; (**C**): plant height in different RMs; (**D**): the total fresh weight in different RMs. Note: Different letters on the bars indicate significant differences. NS, *p* > 0.05; *, *p* < 0.05; **, *p* < 0.01; ***, *p* < 0.001.

For the rooting medium, the different basic media (1/2 MS medium and WPM) had significant effects on the plant height, primary root length and total fresh weight and had no significant effect on the root number after 45 days of cultivation (Figure 6). The applications of hormones (IBA and NAA) had significant effects on the measured parameters (Figure 6). 

**Figure 7:**
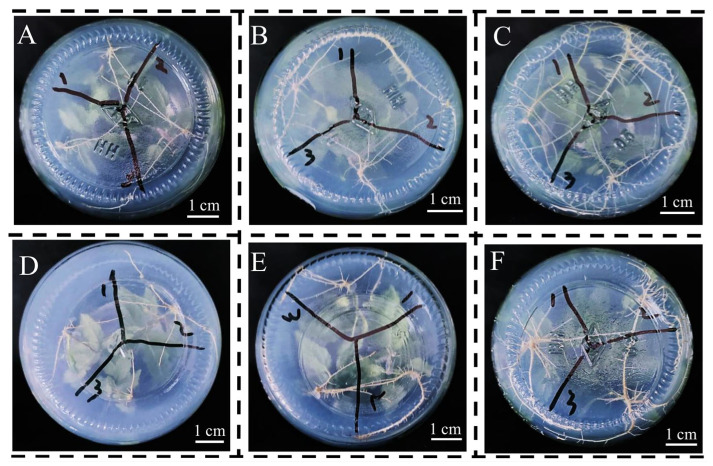
The root systems of gray poplar in different rooting medium after 45 days. (**A**): RM1 (1/2 MS); (**B**): RM2 (1/2 MS + 0.25 mg L^−1^ IBA); (**C**): RM3 (1/2 MS + 0.10 mg L^−1^ NAA); (**D**): RM4 (WPM); (**E**): RM5 (WPM + 0.25 mg L^−1^ IBA); (**F**): RM6 (WPM + 0.10 mg L^−1^ NAA).

For the root number, the average root number in 1/2 MS medium supplemented with NAA was the highest than those in other two 1/2 MS media, with an average root number of four (Figure 6A and Figure 7). Supplementation with NAA in the WPM also significantly increased the root numbers more compared to that of the hormone-free WPM, with an average of 4.2 roots (Figure 6A and Figure 7). Under hormone-free conditions, the primary root lengths of the plants in 1/2 MS medium were significantly longer than those in the WPM; however, supplementation with IBA and NAA could promote primary root growth in the WPM, but could not in 1/2 MS medium (Figure 6B and Figure 7).

The hormones (IBA and NAA) significantly increased plant height by ca. 20% in 1/2 MS medium compared to that of the hormone-free medium; however, only the NAA significantly promoted plant height in the WPM (Figure 6C). The average plant height in the WPM supplemented with NAA was 5.7 cm, which was significantly higher than those in the IBA and hormone-free WPM media (Figure 6C). The results show that both IBA and NAA could promote the plant height in 1/2 MS medium and the WPM, and the effect of the NAA was larger. For plant fresh weight, both the IBA and NAA could significantly increase the accumulation of total fresh weight in both 1/2 MS medium and the WPM, and the NAA was more effective than IBA in both 1/2 MS medium and the WPM (Figure 6D). For instance, compared to the hormone-free medium, supplementation with NAA in 1/2 MS medium and the WPM increased the fresh weight by 166.7% and 145.5%, respectively (Figure 6D).

## 4. Discussion

It was found that the regeneration rates of the direct regeneration system were much higher, and the cycle of inducing adventitious shoots could be shortened by 2–6 weeks. The concentrations of KT in CIM1 and CIM2 were different, but the callus induction rates were both close to 100% (Table 1), which is consistent with the results of Song et al. [44]. CIM2 was more suitable for callus induction for gray poplar in this study. In this experiment, the period of adventitious shoot induction using SIM1 was about 2 months long, while the time taken to grow adventitious shoots using SIM2 was about 3–4 months. The time taken to grow adventitious shoots using SIM1 was shorter, indicating that 0.02 mg L^−1^ TDZ was more suitable for adventitious shoots induction from calluses than the combination of 0.50 mg L^−1^ 6-BA and 0.05 mg L^−1^ NAA in WPM. Low concentrations of TDZ could promote the growth of calluses, the proliferation and regeneration of shoots, but could not promote adventitious shoot elongation. Prolonged exposure to the TDZ-containing medium can lead to vitrification and even inhibit bud differentiation [30], which may be one of the reasons why SIM3 is better than SIM4.

The regeneration abilities of different poplar tissues differ. The regeneration rate of young leaves was higher than that of the old leaves, indicating that young leaves have greater potential for shoot regeneration than old leaves do (Table 1). Young tissues may show a strong metabolism under the promotion of appropriate hormones due to their strong cell viability, which is conducive to differentiation. Young stems and young leaves do not differ significantly in inducing adventitious shoots, but stems are more convenient to use for subsequent experiments (Table 2). Therefore, the young stem segments are more suitable as the explants for regeneration systems, especially for the direct regeneration pathway. Regarding the time cost of obtaining adventitious shoots, the time cost of the indirect regeneration pathway was 2–4 months, while the time taken to use the direct regeneration pathway could be shortened to 1–2 months. Therefore, direct regeneration methods are more effective for inducing adventitious shoots, as they involve a lower time cost.

In plant tissue cultures, plant hormones are usually used for maintaining the stable growth of plants. Different genotypes and tissues have different responses to hormone treatments. When 0.10 mg L^−1^ IBA, 0.05 mg L^−1^ 6-BA and their combination were used, there were no significant effects on the height of the elongated adventitious shoots, but application of 0.05 mg L^−1^ 6-BA significantly promoted the number of elongated adventitious shoots by 61.9% than those of the other two hormone recipes (Figure 5). Among the hormones used in the SEMs, 0.1 mg L^−1^ NAA with MS medium was more effective than the other tested SEMs on the number of elongated adventitious shoots (Figure 5).

Although most poplar species could efficiently induce adventitious root growth in a hormone-free medium [31], supplementation with IBA and NAA in 1/2 MS medium and WPM could more significantly improve the plants’ growth compared to that of using a hormone-free medium alone (Figure 6 and Figure 7). The plants from IBA- or NAA-containing media had higher plant height and total fresh weight with more vigorous root systems (Figure 6 and Figure 7). Moreover, the plants in the 1/2 MS medium with 0.10 mg L^−1^ NAA had much more lateral roots than those in the other RMs (Figure 7), suggesting that NAA could promote lateral root development, thereby contributing to plant growth. Taken together, these data indicate that the NAA is much more effective for promoting root growth than IBA is for gray poplar. The results also suggest that 1/2 MS medium is better than the WPM for gray poplar growth in terms of plant height, root number and fresh weight (Figure 6 and Figure 7).

## 5. Conclusions

Both direct and indirect regeneration pathways can induce adventitious shoots, but the direct regeneration pathway obviously could save a lot of time. In this study, the optimal direct differentiation medium was SIM3 (MS medium supplemented with 0.20 mg L^−1^ 6-BA, 0.10 mg L^−1^ IBA and 0.001 mg L^−1^ TDZ) for gray poplar, and stem segments can be used as explants for regeneration. For the indirect regeneration pathway, the wounded leaves cultured in CIM2 (WPM containing 0.10 mg L^−1^ KT and 1.00 mg L^−1^ 2,4-D) could be a better choice for callus induction. The optimal callus differentiation medium was SIM1 (WPM supplemented with 0.02 mg L^−1^ TDZ). The optimal shoot elongation medium for adventitious shoots was SEM6 (MS with 0.10 mg L^−1^ NAA), and RM3 (1/2 MS medium with 0.10 mg L^−1^ NAA) showed the best performance for rooting, and later, shoot growth. The tissue culture plants obtained in the experiment were not transplanted into the matrix for domestication, and the survival rate needs to be further confirmed. In conclusion, SIM3, SEM6 and RM3 are recommended for a direct regeneration pathway using stems as explants, and CIM2, SIM1, SEM6 and RM3 are suggested for an indirect regeneration pathway using wounded leaves as explants.

## Figures and Tables

**Table 1:** Callus induction and differentiation ratios.

Types of Medium	Explant	Callus Induction Rate/%		Adventitious Shoot Induction Ratio/%	
CIM1 and SIM1	Old leaves	15/15	100.0	1/14	7.1
	Old stems	13/14	92.9	4/30	13.3
	Young leaves	11/11	100.0	8/36	22.2
	Young stems	16/20	80.0	13/31	41.9
CIM2 and SIM2	Old leaves	33/33	100.0	15/72	20.8
	Old stems	12/14	85.7	0	0.0
	Young leaves	49/49	100.0	29/68	42.7
	Young stems	16/16	100.0	0	0.0

**Table 2:** Direct adcventitious shoot induction for different plant tissues.

Types of Medium	Explant	Number of Days to Induce Adventitious Shoots/d	Adventitious Shoot Differentiation Rate/%	
SIM3	Old leaves	45	20/20	100.0
	Old stems	45	19/19	100.0
	Young leaves	45	19/19	100.0
	Young stems	45	18/19	94.7
SIM4	Old leaves	52	56/56	100.0
	Old stems	52	31/31	100.0
	Young leaves	52	50/50	100.0
	Young stems	52	24/25	96.0

## Data Availability

Not applicable.

## References

[1] Luo J., Zhou J.-J., Masclaux-Daubresse C., Wang N., Wang H., Zheng B. (2019). Morphological and physiological responses to contrasting nitrogen regimes in *Populus cathayana* is linked to resources allocation and carbon/nitrogen partition. Environ. Exp. Bot..

[2] Shi W., Zhou J., Li J., Ma C., Zhang Y., Deng S., Yu W., Luo Z.-B. (2021). Lead exposure-induced defense responses result in low lead translocation from the roots to aerial tissues of two contrasting poplar species. Environ. Pollut..

[3] Luo J., Liang Z., Wu M., Mei L. (2019). Genome-wide identification of *BOR* genes in poplar and their roles in response to various environmental stimuli. Environ. Exp. Bot..

[4] Luo J., Zhou J.-J. (2019). Growth performance, photosynthesis, and root characteristics are associated with nitrogen use efficiency in six poplar species. Environ. Exp. Bot..

[5] Li H., Li M., Luo J., Cao X., Qu L., Gai Y., Jiang X., Liu T., Bai H., Janz D. (2012). N-fertilization has different effects on the growth, carbon and nitrogen physiology, and wood properties of slow-and fast-growing *Populus* species. J. Exp. Bot..

[6] Nilsson O., Aldén T., Sitbon F., Anthony Little C., Chalupa V., Sandberg G., Olsson O. (1992). Spatial pattern of cauliflower mosaic virus 35S promoter-luciferase expression in transgenic hybrid aspen trees monitored by enzymatic assay and non-destructive imaging. Transgenic Res..

[7] Wei F., Zhao F.-f., Tian B.-m. (2017). In vitro regeneration of *Populus tomentosa* from petioles. J. For. Res..

[8] Wang H., Wang C., Liu H., Tang R., Zhang H. (2011). An efficient *Agrobacterium*-mediated transformation and regeneration system for leaf explants of two elite aspen hybrid clones *Populus alba* × *P. berolinensis* and *Populus davidiana* × *P. bolleana*. Plant Cell Rep..

[9] Tuskan G.A., Difazio S., Jansson S., Bohlmann J., Grigoriev I., Hellsten U., Putnam N., Ralph S., Rombauts S., Salamov A. (2006). The genome of black cottonwood, *Populus trichocarpa* (Torr. & Gray). Science.

[10] Jansson S., Douglas C.J. (2007). *Populus*: A model system for plant biology. Annu. Rev. Plant Biol..

[11] Cheng Z., Zhang X., Zhao K., Yao W., Li R., Zhou B., Jiang T. (2019). Over-expression of *ERF38* gene enhances salt and osmotic tolerance in transgenic poplar. Front. Plant Sci..

[12] Wang S., Chen Q., Wang W., Wang X., Lu M. (2005). Salt tolerance conferred by over-expression of *OsNHX1* gene in Poplar 84K. Chin. Sci. Bull..

[13] Yu L., Xiong D., Han Z., Liang Y., Tian C. (2019). The mitogen-activated protein kinase gene *CcPmk1* is required for fungal growth, cell wall integrity and pathogenicity in *Cytospora chrysosperma*. Fungal Genet. Biol..

[14] Liu B., Shang X., Zhang X., Shao W., Ren L., Li G., Zhu M., Wang R. (2023). In vitro regeneration and *Agrobacterium*-mediated genetic transformation of *Caragana korshinskii*. For. Res..

[15] Zhou T., Lin Y., Lin Y., Luo J., Ding J. (2022). Regeneration and *Agrobacterium*-mediated genetic transformation of twelve *Eucalyptus* species. For. Res..

[16] Gözükirmizi N., Bajroviç K., İpekçi Z., Boydak M., Akalp T., Tunçtaner K., Balkan H., Tanrıyar H., Çalıkoėlu M., Oėraş T. (1998). Genotype differencies in direct plant regeneration from stem explants of *Populus tremula* in Turkey. J. For. Res..

[17] Ferreira S., Batista D., Serrazina S., Pais M.S. (2009). Morphogenesis induction and organogenic nodule differentiation in *Populus euphratica* Oliv. leaf explants. Plant Cell Tissue Organ Cult..

[18] Aggarwal G., Gaur A., Srivastava D.K. (2015). Establishment of high frequency shoot regeneration system in Himalayan poplar (*Populus ciliata* Wall. ex Royle) from petiole explants using Thidiazuron cytokinin as plant growth regulator. J. For. Res..

[19] Maheshwari P., Kovalchuk I. (2011). Efficient shoot regeneration from internodal explants of *Populus angustifolia*, *Populus balsamifera* and *Populus deltoids*. New Biotechnol..

[20] Hai G., Jia Z., Xu W., Wang C., Cao S., Liu J., Cheng Y. (2016). Characterization of the *Populus PtrCesA4* promoter in transgenic *Populus alba* × *P. glandulosa*. Plant Cell Tissue Organ Cult..

[21] Zhu T., Wang J., Hu J., Ling J. (2022). Mini review: Application of the somatic embryogenesis technique in conifer species. For. Res..

[22] Yan X., Wang K., Zheng K., Zhang L., Ye Y., Qi L., Zhu M. (2023). Efficient organogenesis and taxifolin production system from mature zygotic embryos and needles in larch. For. Res..

[23] Guo M., Yu Q., Li D., Xu K., Di Z., Zhang Y., Yu Y., Zheng J., Zhang Y. (2023). In vitro propagation, shoot regeneration, callus induction, and suspension from lamina explants of *Sorbus caloneura*. For. Res..

[24] Zheng L., Yang J., Chen Y., Ding L., Wei J., Wang H. (2021). An improved and efficient method of *Agrobacterium* syringe infiltration for transient transformation and its application in the elucidation of gene function in poplar. BMC Plant Biol..

[25] Jia Z., Sun Y., Yuan L., Tian Q., Luo K. (2010). The chitinase gene (*Bbchit1*) from *Beauveria bassiana* enhances resistance to *Cytospora chrysosperma* in *Populus tomentosa* Carr. Biotechnol. Lett..

[26] Wen S.S., Ge X.L., Wang R., Yang H.F., Bai Y.E., Guo Y.H., Zhang J., Lu M.Z., Zhao S.T., Wang L.Q. (2022). An efficient *Agrobacterium*-mediated transformation method for hybrid poplar 84K (*Populus alba* × *P. glandulosa*) using calli as explants. Int. J. Mol. Sci..

[27] Movahedi A., Zhang J., Amirian R., Zhuge Q. (2014). An efficient *Agrobacterium*-mediated transformation system for poplar. Int. J. Mol. Sci..

[28] Muhr M., Prüfer N., Paulat M., Teichmann T. (2016). Knockdown of strigolactone biosynthesis genes in *Populus* affects *BRANCHED 1* expression and shoot architecture. New Phytol..

[29] Zhou H., Song X., Wei K., Zhao Y., Jiang C., Wang J., Tang F., Lu M. (2019). Growth-regulating factor 15 is required for leaf size control in *Populus*. Tree Physiol..

[30] Li S., Zhen C., Xu W., Wang C., Cheng Y. (2017). Simple, rapid and efficient transformation of genotype Nisqually-1: A basic tool for the first sequenced model tree. Sci. Rep..

[31] Luo J., Nvsvrot T., Wang N. (2021). Comparative transcriptomic analysis uncovers conserved pathways involved in adventitious root formation in poplar. Physiol. Mol. Biol. Plants.

[32] Barkla B.J., Vera-Estrella R., Pantoja O. (2014). Growing Arabidopsis in vitro: Cell suspensions, in vitro culture, and regeneration. Arabidopsis Protocols.

[33] Maheshwari P., Kovalchuk I. (2016). *Agrobacterium*-mediated stable genetic transformation of *Populus angustifolia* and *Populus balsamifera*. Front. Plant Sci..

[34] Tsai C.-J., Podila G.K., Chiang V.L. (1994). *Agrobacterium-mediated* transformation of quaking aspen (*Populus tremuloides*) and regeneration of transgenic plants. Plant Cell Rep..

[35] Han X., Ma S., Kong X., Takano T., Liu S. (2013). Efficient *Agrobacterium-mediated* transformation of hybrid poplar *Populus davidiana* Dode × *Populus bollena* Lauche. Int. J. Mol. Sci..

[36] Coleman G.D., Ernst S.G. (1989). In vitro shoot regeneration of *Populus deltoides*: Effect of cytokinin and genotype. Plant Cell Rep..

[37] Souza J., Tomaz M., Arruda S., Demétrio C.G.B., Venables W., Martinelli A.P. (2011). Callus sieving is effective in improving synchronization and frequency of somatic embryogenesis in *Citrus sinensis*. Biol. Plant..

[38] Huang Z., Xu C., Li Y., Wang P., Li Y., Kang X. (2015). Induction of somatic embryogenesis by anther-derived callus culture and plantlet ploidy determination in poplar (*Populus* × beijingensis). Plant Cell Tissue Organ Cult..

[39] Balestrazzi A., Carbonera D., Confalonieri M. (2000). *Agrobacterium tumefaciens*-mediated transformation of elite white poplar (*Populus alba* L.) and regeneration of transgenic plants. J. Genet. Breed..

[40] Luo J., Shi W., Li H., Janz D., Luo Z.-B. (2016). The conserved salt-responsive genes in the roots of *Populus* × *canescens* and *Arabidopsis thaliana*. Environ. Exp. Bot..

[41] Li Z., Deng S., Zhu D., Wu J., Zhou J., Shi W., Fayyaz P., Luo Z.-B., Luo J. (2023). Proteomic reconfigurations underlying physiological alterations in poplar roots in acclimation to changing nitrogen availability. Environ. Exp. Bot..

[42] He J., Li H., Luo J., Ma C., Li S., Qu L., Gai Y., Jiang X., Janz D., Polle A. (2013). A transcriptomic network underlies microstructural and physiological responses to cadmium in *Populus* × *canescens*. Plant Physiol..

[43] Filichkin S., Meilan R., Busov V., Ma C., Brunner A., Strauss S. (2006). Alcohol-inducible gene expression in transgenic *Populus*. Plant Cell Rep..

[44] Song J., Lu S., Chen Z.-Z., Lourenco R., Chiang V.L. (2006). Genetic transformation of *Populus trichocarpa* genotype Nisqually-1: A functional genomic tool for woody plants. Plant Cell Physiol..

